# Increased incidence of syphilis in men who have sex with men and risk management strategies, Germany, 2015

**DOI:** 10.2807/1560-7917.ES.2016.21.43.30382

**Published:** 2016-10-27

**Authors:** Klaus Jansen, Axel J. Schmidt, Jochen Drewes, Viviane Bremer, Ulrich Marcus

**Affiliations:** 1 Robert Koch Institute, Unit for HIV/AIDS, STI and blood-borne infections, Berlin, Germany; 2 London School of Hygiene & Tropical Medicine (LSHTM), London, United Kingdom; 3 Swiss Federal Office of Public Health (FOPH), Bern, Switzerland; 4 Free University Berlin, Institute for Prevention and Psychosocial Health Research, Berlin, Germany

**Keywords:** syphilis, men who have sex with men – MSM, epidemiology, surveillance, sexual behaviour

## Abstract

In Germany, the number of reported syphilis cases increased between 11% and 22% per year between 2010 and 2014. We analysed syphilis surveillance data and data of four behavioural surveys on men who have sex with men (MSM) in Germany (2003, 2007, 2010, 2013) to assess if this rise is ongoing and to find possible explanations for it. Syphilis notifications increased in 2015 by 19% to a total of 6,834. This was mainly due to increasing notifications in MSM of all age groups in larger German cities. Data from the behavioural surveys on MSM in Germany showed a simultaneous increase of selective condom use as HIV-status-bases risk management strategy and the number of syphilis cases. MSM diagnosed with HIV reported condomless anal intercourse with non-steady partners more frequent than MSM not diagnosed with HIV or untested for HIV, but the latter also reported higher frequencies of this behaviour in the more recent surveys. Transmission in HIV-positive MSM probably plays an important, but not exclusive role, for the syphilis dynamics in Germany. A risk adapted routine screening for sexually active MSM and potentially innovative approaches to increase early screening and treatment of syphilis such as internet counselling, home sampling, home testing and broadening venue-based (rapid) testing, should be critically evaluated to effectively reduce syphilis infections.

## Introduction

Syphilis incidence among men who have sex with men (MSM) has been on the rise globally during the last years. Especially in western countries, sharp increases in numbers of syphilis infections were observed [1-4]. In Europe, the syphilis incidence was 5.1 cases/100,000 inhabitants overall, with distinct differences between countries, probably due to the differences in the notification systems, completeness of data and healthcare structures [3]. Since 2009, the syphilis incidence increased in Europe in men, especially in western European countries, while the incidence decreased in women concurrently. In Germany, the number of reported syphilis cases doubled between 2001 and 2004 to over 3,000 per year and remained mainly stable until 2009. Between 2010 and 2014, the number increased between 11% and 22% per year [5].

High rates of bacterial sexually-transmitted infections (STIs) including syphilis are reported for MSM coinfected with HIV from many countries, e.g. Australia, Canada, England, Germany, and Spain [6-10]. We discuss reasons for the increasing syphilis incidence in MSM, in particular the increase in risky sexual behaviour, such as a higher frequency of condomless sexual intercourse, while applying HIV serostatus knowledge-based risk management strategies, particularly HIV-serosorting [11-16].

Syphilis is a STI caused by *Treponema pallidum*. It has different stages of disease (primary, secondary, latent, and tertiary syphilis), of which especially the first and second stages are highly infectious. Syphilis can lead to severe sequelae such as serious cardiovascular or neurological impairments and also death, and increases the risk of HIV acquisition and transmission [17,18]. As congenital syphilis, *T. pallidum* can also be transmitted to a fetus during pregnancy and can cause severe health impairments of the newborn, including premature delivery and stillbirth. Syphilis can still be treated effectively with penicillin [17].

To assess the epidemiological dynamics of syphilis in Germany and to shape appropriate public health interventions, we analysed data of the mandatory syphilis notification system reported between 2001 and 2015. Additionally, we analysed data of four waves of a behavioural survey among MSM in Germany to assess potential changes in relevant sexual behaviours.

## Methods

### Mandatory syphilis notification

In Germany, syphilis diagnoses have been notified anonymously on the basis of the Protection against Infection Act in Germany [19] since 2001 by laboratories, with physicians inserting relevant clinical information. Syphilis cases are defined as cases diagnosed by direct detection of *T. pallidum* by microscopic or histological examination OR a positive screening test and a confirmation test (a combination of *T. pallidum* particle agglutination test (TPPA), *T. pallidum* haemoagglutination test (TPHA), Immuno-Assay, fluorescence *Treponema* antibody absorption test (FTA-ABS), Immunoblot) AND venereal disease research laboratory (VDRL)/rapid plasma reagin (RPR) activity or IgM antibodies OR clinical information consistent with syphilis [17].

Potential double notifications were identified by comparing cases by demographic data, diagnosis date, antibody titres, and clinical information. We analysed syphilis cases by year of diagnosis, age, sex, area of residence, and transmission group.

### Behavioural surveys

Self-reported data on sexual risk behaviours and diagnoses of HIV and syphilis among MSM were collected during four waves of a behavioural MSM survey in 2003, 2007, 2010, and 2013. Survey participants were recruited exclusively online in the 2010 and 2013 surveys, and by a combination of print questionnaires distributed by gay magazines and online questionnaires in the 2003 and 2007 surveys. The methods and the results of this survey have been published elsewhere in German [20-23]. These surveys are part of the HIV behavioural surveillance in Germany implemented in the late 1980s [22]. Although they use the same or very similar questions, their comparability is restricted, mainly due to the different recruitment methods. Recruitment bias affected information on age, city size, and sexual identity. This is why we restricted the analysis to a subgroup of men self-identified as gay, aged 30–44 years, and living in cities with more than 500,000 inhabitants (in descending order according to the number of inhabitants: Berlin, Hamburg, Munich, Cologne, Frankfurt am Main, Stuttgart, Dusseldorf, Dortmund, Essen, Bremen, Leipzig, Dresden, Hanover, Nuremberg). This subgroup is less affected by the change in recruitment methods. The sample sizes of the surveys were: 4,750 in 2003,  8,170 in 2007, 54,387 in 2010, and 16,734 in 2013. The subgroup of gay-identified men aged 30–44 years and living in cities with more than 500,000 inhabitants consisted of 1,039 (22%) men in 2003, 1,315 (16%) in 2007, 8,242 (15%) in 2010, and 1,547 (9.2%) in 2013.

We analysed trends in condomless anal intercourse (cAI) with *steady* and *non-steady* partners in the previous 12 months (scAI respectively nscAI), and with partners of *unknown* HIV status (ucAI), also stratified by HIV status, as well as the proportion of MSM getting tested for HIV in the previous 12 months, to explore the increasing syphilis transmission among MSM. Data on syphilis testing were only collected in 2010 and 2013 in the behavioural surveys.

Data were analysed using descriptive statistics.

## Results

### Data from mandatory syphilis notification

**Figure 1:**
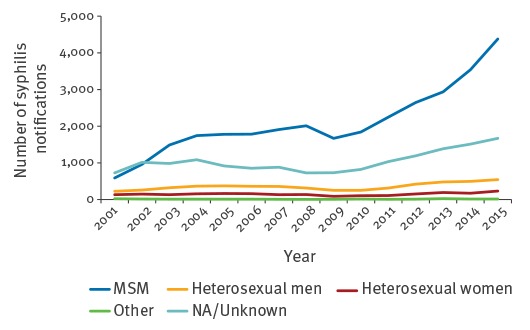
Number of syphilis notifications, by transmission group, Germany, 2001–2015

MSM: men who have sex with men; NA: not available.

As at 1 March 2016, 54,747 newly diagnosed cases of syphilis had been notified in Germany between 1 January 2001 and 31 December 2015, with cases increasing since 2010 (Figure 1). In 2015, 6,834 cases were reported, corresponding to a 19.4% increase compared with 2014. Incidence was 8.5 per 100,000 inhabitants overall, with highest incidences above 20.0 mainly in larger German cities such as Berlin (39.0), Cologne (35.6), Munich (30.0), Frankfurt am Main (29.5), Dusseldorf (26.6), Leipzig (23.7), Hamburg (21.4) and Stuttgart (20.4). They were especially high in Berlin inner city areas with 62.8–117.8/100,000 inhabitants. Notified cases increased in 14 of 16 German federal states in 2015.

Men accounted for 93.8% of cases in 2015 (n = 6,834). The transmission route was reported for 75.6% of cases (n = 5,166); of these, 84.7% occurred in MSM, 15.0% among heterosexual persons, and 0.3% were acquired through other routes of transmission.

**Figure 2:**
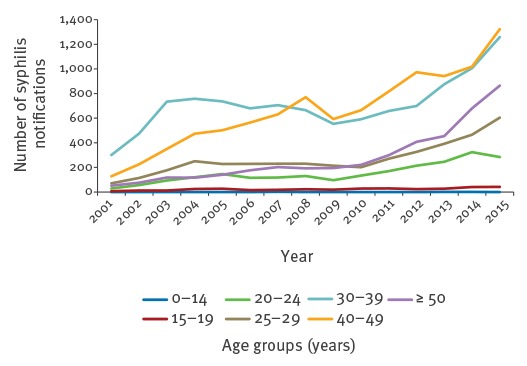
Number of syphilis notifications in MSM, by age group, Germany, 2001–2015

In 2015, 84.9% of MSM diagnosed with syphilis originated from Germany (n = 3,758), and 95.6% of syphilis cases in MSM were reportedly acquired in Germany (n = 3,981). Since 2008, at least half of the syphilis cases among MSM have been diagnosed in men aged 40 years and older (n = 54,744, Figure 2). Since 2007, the proportion of MSM diagnosed in primary or secondary stages of disease has remained between 60.4% and 67.7% (n = 40,005). Since 2006, physicians provided information on re-infection for 64.8% of all reported syphilis cases: between 40.4% and 50.9% of all syphilis cases reported in MSM were categorised as re-infection (n = 21,761).

MSM: men who have sex with men.

### Data from behavioural surveys

In the analysed subsample of men self-identified as gay, aged 30–44 years and living in cities with more than 500,000 inhabitants, the HIV prevalence was 15.9% in 2003 (n = 1,039), 14.9% in 2007 (n = 1,315), 16.9% in 2010 (n = 8,242), and 22.3% in 2013 (n = 1,547).

**Figure 3:**
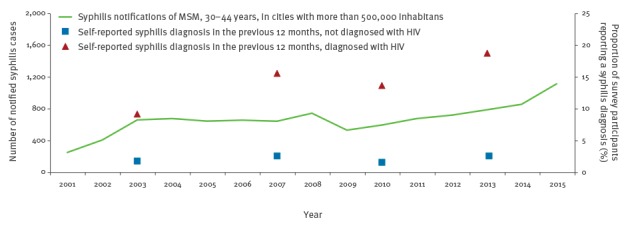
Number of syphilis notifications and self-reported syphilis diagnoses in MSM aged 30–44 years and living in cities with more than 500,000 inhabitants, Germany, 2001–2015

The trend of self-reported syphilis cases was similar to the trend in syphilis notifications (Figure 3); the increasing trend was almost entirely based on respondents with HIV. Between 2003 and 2013, the proportion of MSM diagnosed with HIV (n = 1,934) reporting newly diagnosed syphilis, increased from 9.3% to 19.0%, while the proportion of MSM not diagnosed with HIV (n = 9,397) and self-reporting a recent syphilis diagnosis, fluctuated between 1.7% and 2.7%.

MSM: men who have sex with men.

During the survey years, the following numbers of MSM participated in the behavioural surveys: 2003 (n = 1,039), 2007 (n = 1,315), 2010 (n = 8,242), 2013 (n = 1,547).

The total number of syphilis notifications fulfilling the inclusion criteria was 10,120.

**Figure 4:**
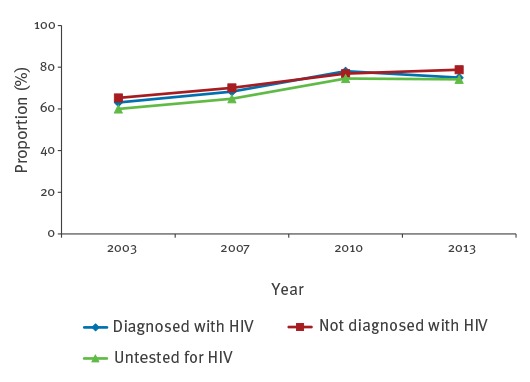
Proportions of survey respondents self-reporting condomless anal intercourse with steady partners (scAI) in the previous 12 months, by HIV status of respondents, MSM aged 30–44 years and living in cities with more than 500,000 inhabitants, Germany, 2003–2013

HIV-testing in the previous 12 months increased from 32.8% (2003) to 34.8% (2007), 41.5% (2010) and 48.4% (2013). Among all MSM reporting a syphilis diagnosis in the previous 12 months (2003: 29; 2007: 60; 2010: 306; 2013: 58), the proportion of MSM diagnosed with HIV increased from 48.3% (2003) to 50.0% (2007), 62.4% (2010), and 69.0% (2013). Partly, sexual behaviour differed considerably by self-reported HIV status. The proportions of scAI were high and increasing for MSM independently of their HIV status (Figure 4).

MSM: men who have sex with men.

During the survey years, the following numbers of MSM participated in the behavioural surveys: 2003 (n = 472), 2007 (n = 624), 2010 (n = 3,272), 2013 (n = 586).

**Figure 5:**
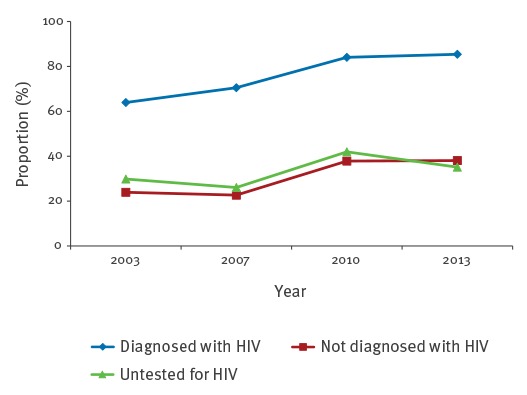
Proportions of survey respondents self-reporting condomless anal intercourse with non-steady partners (nscAI) in the previous 12 months, by HIV status of respondents, MSM aged 30–44 years and living in cities with more than 500,000 inhabitants, Germany, 2003–2013

The proportion of MSM diagnosed with HIV and reporting nscAI (n = 1,334) was more than double compared with the respective proportion of MSM not diagnosed with (n = 1,857) or not tested for HIV (n = 272) (Figure 5). Reporting of nscAI increased over the years for all those groups, with the exception of 2013, for MSM untested for HIV. We found the strongest increase with 59% between 2003 and 2013 for MSM not diagnosed with HIV.

MSM: men who have sex with men.

During the survey years, the following numbers of MSM participated in the behavioural surveys: 2003 (n = 740), 2007 (n = 870), 2010 (n = 5,132), 2013 (n =  966).

**Figure 6:**
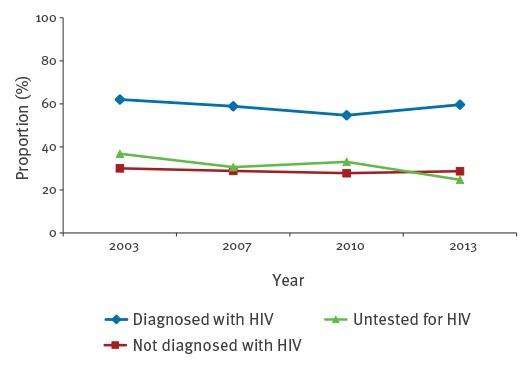
Proportions of survey respondents self-reporting condomless anal intercourse with partners of unknown HIV status (ucAI) in the previous 12 months, by HIV status of respondents, MSM aged 30–44 years and living in cities with more than 500,000 inhabitants, Germany, 2003–2013

With slight decreases, the proportions of MSM reporting ucAI stayed stable between 2003 and 2013 (Figure 6). MSM diagnosed with HIV reported ucAI more frequently than MSM not diagnosed with or not tested for HIV.

MSM: men who have sex with men.

During the survey years, the following numbers of MSM participated in the behavioural surveys: 2003 (n = 708), 2007 (n = 876), 2010 (n =  4,272), 2013 (n =  961).

Testing for syphilis was reported much more frequently by survey respondents diagnosed with HIV compared with survey respondents not diagnosed with HIV. The proportion of respondents diagnosed with HIV reporting at least one syphilis test in the previous 12 months increased in our subsample from 80% in 2010 to 88.5% in 2013. Among respondents tested for, but not diagnosed with HIV this proportion increased from 33.5% in 2010 to 35.8% in 2013. The proportion of MSM reporting a syphilis test in the previous 12 months and never tested for HIV decreased from 5.7% in 2010 to 3.2% in 2013.

## Discussion

We found an accelerating increase of syphilis notifications in Germany since 2010. This increase was mainly due to a rise in the number of newly diagnosed cases in MSM, acquired domestically. The epidemic is mostly concentrated in larger cities and more densely inhabited regions of Germany, where the proportion of MSM is higher [24,25]. Berlin as a centre of sex tourism for MSM [26] was heavily affected. The increase applied to MSM in all age groups, and was strongest in older age groups in terms of absolute numbers.

The analysis of survey data on sexual behaviours of MSM in Germany provided an indication that changes in sexual behaviours of MSM taking place during the last years may have played an important role in the increase in the number of syphilis cases. We observed a coincident increase of HIV-status-based risk management (selective condom use, ‘serosorting’) and increasing syphilis cases. cAI with steady partners (scAI) has become increasingly common, regardless of HIV status. Apart from scAI, we found distinct differences between MSM diagnosed and not diagnosed with HIV. If the partner was a non-steady partner, cAI was more commonly reported by MSM diagnosed with HIV than if the partner was a steady partner. MSM not diagnosed with or untested for HIV less commonly reported cAI with non-steady partners, but the proportion reporting cAI increased in the more recent surveys. We found almost no changes over time in ucAI, both for MSM diagnosed with HIV and for those not diagnosed with HIV; only among MSM untested for HIV, the proportion reporting this behaviour decreased over time. Even though the proportion of MSM diagnosed with HIV and reporting ucAI was much higher than that of MSM not diagnosed with HIV or untested for HIV, it is most likely that MSM diagnosed with HIV were effectively treated with antiretrovirals, and thus not infectious for HIV, and therefore not compelled to discuss their own HIV status or that of their sex partner.

There was an increase in cAI in MSM with both steady and non-steady partners. However, we believe that for the increasing syphilis incidence, the increase in cAI with non-steady partners is much more relevant because having different sex partners is one of the major risk factors for acquiring syphilis among MSM [26].

An increase in cAI was also reported from the United States (US) behavioural surveillance system in MSM [27]. However, the authors’ interpretation did not link this to serosorting, because the increase appeared to be independent from the HIV test status and was also observed among MSM never tested. We can confirm from our data that increased reporting of cAI can be observed independently of the HIV testing history. However, we argue that MSM never tested for HIV are participating in serostatus communication, most of them assuming that they are not infected. This assumption was supported by our data since the proportion of men reporting cAI with partners of unknown HIV status was stable or even declined, based on a question which was not used in the US surveillance. This question (‘Did you have anal intercourse without a condom with a partner with unknown HIV test result?’) may also be negated by MSM who have never been tested, but assume or are told by their partners that these partners are HIV-negative. It is probable that MSM in this group practice serosorting similarly to MSM being HIV-negative. In any case, there is evidence from MSM behavioural surveys that a fraction of men never tested for HIV report telling their partners being HIV-negative [26].

An increase in syphilis cases was seen in both first and second generation surveillance. Survey data showed that syphilis among MSM seemed to be largely and increasingly concentrated among MSM with diagnosed HIV. However, variation in recruitment methods, sample sizes and sample composition of the MSM surveys limit the generalisability of behavioural trends to the overall MSM population in Germany. Although no increase in the proportion of self-reported syphilis diagnoses could be observed among survey respondents without diagnosed HIV, we would like to point out that the absolute numbers of syphilis cases in this population could still be higher than the number of syphilis infections among MSM living with HIV.

Survey participants could not be proven representative either for all MSM diagnosed with HIV or for the total MSM population, and differing self-selection biases may distort the composition of the survey respondents. If we ignore such unknown biases and extrapolate the observed syphilis incidence among survey respondents diagnosed with HIV and not diagnosed with HIV, to the estimated population of all MSM diagnosed with HIV (n = 42,000 at the end of 2013 [28]) and all MSM not diagnosed with HIV in Germany (n = 700,000 [29]), we would have to expect more cases among HIV-negative than among HIV-positive MSM. We hypothesise that the susceptible population connected to sexual networks created by online- and smartphone-dating might have expanded over the recent years [30]. This could explain increasing numbers of syphilis cases among HIV-negative MSM without an increase in the proportions observed in the surveys. The increasing total number of survey participants over time is compatible with this hypothesis. Molecular epidemiological data would allow for an in-depth analysis of the transmission dynamics of syphilis in Germany and could generate evidence if syphilis infections occurred mainly in core sexual networks of HIV-positive MSM, but these data are not yet available.

Until 2015, the German syphilis notification system provided no data on the HIV status of the reported person. Since 2016, the notification system has been amended and reporting of coinfection with HIV and other STIs has been implemented. This change will enable us to better evaluate the impact of HIV coinfection on the dynamics of syphilis.

About a third of notified cases among MSM were diagnosed with syphilis in late stages of disease, and reinfections were common. This underlines the importance of effective behavioural prevention and broad screening offers for MSM regarding syphilis and other STIs [31]. Consistent condom use independent of HIV status should be promoted for anal intercourse to reduce syphilis transmission. In our subsample from the behaviour surveys, a large majority of MSM diagnosed with HIV have been screened for syphilis in the last 12 months [32]. This does not seem to have a large impact on syphilis incidence in this group. While guidelines have changed and consecutively also screening practices may have changed over time in Germany (so far no direct audits of practices and adherence to guidelines have been conducted), practices are more likely to be influenced by reimbursement rules and concerns rather than by guidelines. In Germany, syphilis testing is easily reimbursable for people diagnosed with HIV through a special reimbursement framework for HIV care while syphilis screening (testing without symptoms) of MSM not diagnosed with HIV may be restricted by reimbursement concerns, in the absence of an official screening programme irrespective of guideline recommendations.

Modelling exercises in Australia and Canada concluded that the frequency of syphilis screening probably needs to be increased to at least biannual screening, in order to achieve an epidemiological impact [33-35]. German guidelines advise for a risk-adapted frequency of screening for MSM [36]. For sexually active MSM especially with changing sex partners, routine screening for syphilis seems to be paramount [37]. To foster this, potentially innovative approaches to increase early screening and treatment such as Internet counselling, home sampling, home testing and broadening venue-based (rapid) testing, should be critically evaluated.
